# TREM1^+^ tumor-associated macrophages secrete CCL7 to promote hepatocellular carcinoma metastasis

**DOI:** 10.1007/s00432-024-05831-1

**Published:** 2024-06-25

**Authors:** Simin Huang, Longguang He, Yufei Zhao, Yuxuan Wei, Qiwen Wang, Yi Gao, Xiaofeng Jiang

**Affiliations:** 1 Department of Hepatobiliary Surgery, The Second Affiliated Hospital of Guangzhou Medical University, Guangzhou, 510260 China; 2 Liver Cancer Center, The Second Affiliated Hospital of Guangzhou Medical University, Guangzhou, 510260 China; 3 Department of Hepatobiliary Surgery II, Zhujiang Hospital, Southern Medical University, Guangdong Guangzhou, 510282 China; 4 Department of Hepatobiliary Surgery, Gaozhou People’s Hospital, Guangdong Gaozhou, 525000 China; 5 Department of Gastrointestinal Surgery, Lab of Surgery, the Second Affiliated Hospital of Guangzhou Medical University, Guangzhou, 510260 China

**Keywords:** TREM1, Tumor-associated macrophages, CCL7, Metastatic HCC

## Abstract

**Purpose:**

Tumor-associated macrophages (TAMs) play a critical role in hepatocellular carcinoma (HCC) progression and metastasis. Systematic investigation of the cross-talk between TAMs and HCC may help in searching for the critical target to guard against HCC metastasis.

**Methods and results:**

Herein, we found that TREM1 highly expressed in HCC tissue by analyzing the data obtain from GEO database. Interestingly, the results indicated that TREM1 was primarily expressed by monocytes. Immune infiltration studies further validated that TREM1 expression was positively related with increased infiltration of macrophages in HCC tissues. In vitro, we observed that TREM1 knockdown significantly abrogated the effect of TAMs in promoting the metastasis and epithelial-mesenchymal transition (EMT) of HCC cells. Additionally, cytokine array detection identified CCL7 as the main responsive cytokine following with TREM1 knockdown in TAMs.

**Conclusion:**

Taken together, our findings strongly suggested that high expression of TREM1 was positively associated with metastasis and poor prognosis of HCC. Furthermore, TAMs expressing TREM1 contribute to EMT-based metastasis through secreting CCL7. These results provide a novel insight into the potential development of targeting the TREM1/CCL7 pathway for preventing metastatic HCC.

**Supplementary Information:**

The online version contains supplementary material available at 10.1007/s00432-024-05831-1.

## Introduction

Hepatocellular carcinoma (HCC) is the predominant type of primary liver cancer, which counts for 90% of cases(Vogel et al. 2022). HCC is the sixth most common cancer worldwide and the third leading cause of cancer-related death(Sung et al. 2021). Unfortunately, most patients with HCC are diagnosed at intermediate or advanced stages with limited effective treatment options available(Fu and Wang 2018). Metastasis is the leading cause of poor prognosis in patients with HCC(Jayachandran et al. 2016). The 5-year survival rate for patients with metastatic HCC is less than 10%(Cabibbo et al. 2010). Therefore, it is crucial to conduct comprehensive research for understanding the underlying mechanisms of metastatic HCC.

Tumor microenvironment (TME) encompasses various components such as tumor cells, surrounding fibroblasts, immune cells, glial cells, interstitial cells, microvessels, and infiltrated biomolecules(Fujita et al. 2020). Among them, immune cells are the important component of the tumor microenvironment (TME) and play a key role in tumor progression(Milo et al. 2018). Specifically, tumor-associated macrophages (TAMs) account for nearly half of a solid tumor mass(Vinogradov et al. 2014). As a highly heterogeneous group of immune cells with diverse phenotypes and functions, TAMs also play a pivotal role in tumor progression(Cheng et al. 2022). The traditional method simply divides TAMs into two subtypes: M1 and M2 types; and M1 subtypes exhibits anti-tumor effects while M2 promotes tumor cell proliferation, metastasis and immune evasion(Cheng et al. 2022). However, the simple classification reduces the accuracy of describing the function of TAMs in regulating tumor progression. Further research is required to establish more detailed classifications within TAMs. Transcriptomic analysis indicated that there may be more differentiated types of TAMs in the tumor microenvironment(Li et al. 2022). Based on different stimuli, a review summarized four different M2 subtypes including M2a, M2b, M2c, and M2d, indicating four different functions of TAMs respectively(Yao et al. 2019). As a highly heterogeneous cell population, TAMs embodied different pro-oncogenic functions with different markers. For example, a study indicated that specifically targeting CD163^+^ TAMs can activate inflammatory monocytes and promote T cell-based immunoclearance against melanoma(Etzerodt et al. 2019). Hence, it is important to decipher the main gene response to poor prognosis of HCC and to explore the main subtypes of TAMs promoting the metastasis of HCC. Moreover, as a simple and convenient strategy, bioinformatics analysis is a good choice to probe the underlying therapeutic targets against metastatic HCC.

In this study, we confirmed that TREM1 was the significant responsive gene that positively correlates with the poor prognosis of HCC. Furthermore, the result indicated that TREM1 mainly expressed by TAMs. It is demonstrated that TREM1 knockdown in THP1-derived TAMs abrogated the effect of TAMs in promoting the migration, invasion, and epithelial-mesenchymal transition (EMT) of HCC cells. More importantly, CCL7 derived from TREM1^+^ TAMs significantly promoted the migration and invasion of HCC. In conclusion, CCL7 derived from TREM1^+^ TAMs can promote hepatocellular carcinoma metastasis. This study identifies TREM1/CCL7 as a novel oncogenic target promoting the metastasis of HCC.

## Materials and methods

### Research framework

**Fig. 1:**
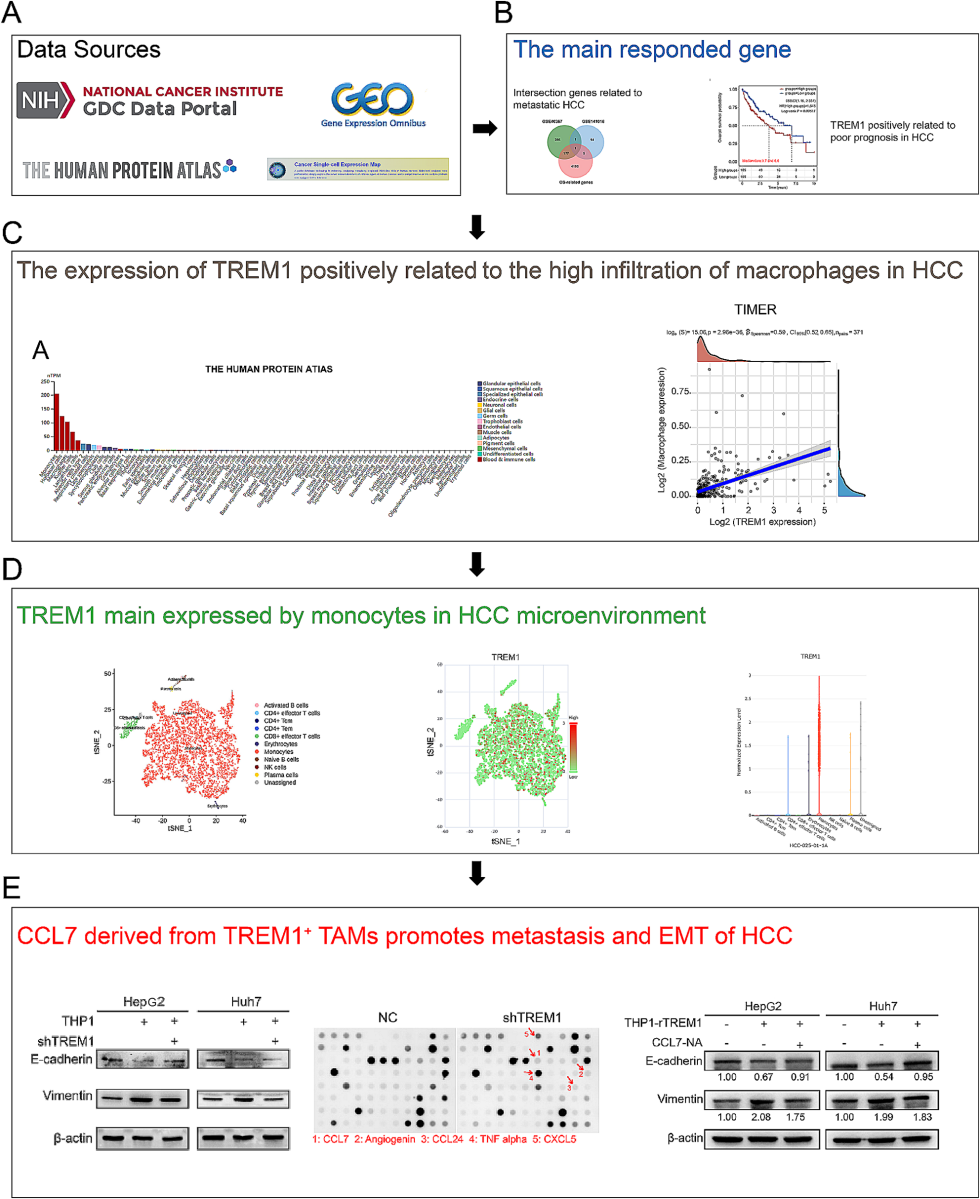
The framework of this research was recorded

Figure 1 showed the framework of this research.

### Data retrieval and analysis

These meaningful data obtained from GEO database (http://www.ncbi.nlm.nih.gov/geo), TCGA database (https://tcga-data.nci.nih.gov/tcga/) were used to detect target genes associated with poor prognosis in patients with HCC. Conveniently, all above data retrieva and analysis were performed in *ACLBI Web-based Tools* (https://www.aclbi.com/) or *THE HUMAN PROTEIN ATIAS* (https://www.proteinatlas.org). GSE40367 and GSE141016 datasets were obtained from the GEO database and MINiML data format were download. Differential expression of mRNA was studied using limma package of R software (R foundation for statistical computing, Version: 3.40.2). “Adjusted P < 0.05 with log2 (fold change) ≥ 1 or log2 (fold change) ≤ -1” was defined as a threshold of differentially expressed genes. RNAseq data (level 3) and related clinical information of 371 patients with HCC were obtained from the TCGA database, and the samples were divided into high-expression groups and low-expression groups according to the median value of TREM1 expression. Moreover, univariate cox regression analysis and log-rank test were used to evaluate the prognostic significance of every single gene according to the expression level, and the target genes positively related to Overall Survival (OS) in patients with HCC were screened. All analysis methods and R packages are implemented using R software.

### Tissue microarray analysis

A commercial microarray (HLivH150CS06, Shanghai Outdo Biotech Company, China) was used to analyze the expression of TREM1 in HCC tissues. TREM1(11791-1-AP, Proteintech) expression in HCC tissues was detected and quantified using immune response score (IRS) method(Huang et al. 2018). Briefly, two experienced pathologists semi-quantitatively scored positive cell percentage (PP%) and staining intensity (SI). The final scores were obtained after the results were multiplied: IRS = PP×SI. When the IRS result falls within the 0–1 range, the expression of the target protein is characterized as minimal or negligible; when it falls within the 2–3 range, that is characterized as weak expression; when it falls within the 4–8 range, that is characterized as medium expression; when it falls within the 9–12 range, that is characterized as strong expression.

### Single-cell sequencing analysis

To identify the target cells mainly expressing TREM1 in HCC microenvironment, single-cell sequencing analysis were performed in *CancerSCEM* (https://ngdc.cncb.ac.cn/cancerscem/). A dataset consisting of 6582 cells was utilized for analysis, with the following parameters employed in the study: resolution set at 1, principal components ranging from 1 to 20, and a threshold of less than 10% for mitochondrial content.

### Venn diagrams

Venn diagrams were created to analyze the difference between several data set using a web-based tool (Bardou et al. 2014) (http://www.bioinformatics.com.cn/).

### Cell culture

The HCC cell line HepG2, Huh7 and THP1 were purchased from the American Type Culture Collection (ATCC, Maryland, USA) and cultured in DMEM medium or 1640 medium (Gibco, Grand Island, NY, USA) supplemented with 1% penicillin and streptomycin and 10% fetal bovine serum (Gibco). The culture of these cells needed to maintain at 37 °C and 5% CO_2_. THP1-derived TAMs were structured via the stimulation with AMP for 24 h, followed by IL-4 for 48 h(Huang et al. 2020).

### Flow cytometry

For M2 types of TAMs population analysis, THP1 cells were stimulated via above methods for TAMs differentiation. The F4/80^+^/CD206^+^ subpopulation was quantified by flow cytometry and defined as M2-TAMs. The commercialized FITC-conjugated F4/80 (BM8, Invitrogen) and PE-conjugated CD206 antibody (685,641, Invitrogen) were purchased. After preparing the single-cell suspension, the cells were incubated with F4/80 and CD206 flow antibodies at thermostat water baths with 37℃, and then the cell precipitation was obtained by centrifugation at 1000 rpm. Subsequently, the cells were resuspended and tested by NovoCyte Advanteon Flow Cytometer (Agilent).

### Plasmid transfection

The commercialized recombinant plasmid and shRNA plasmid of TREM1 as well as non-targeted control plasmid purchased from Vigene Biosciences (WZ Biosciences Inc. China). THP1 cells in logarithmic growth stage were inoculated into 24-well cell culture plates. Cultured medium with serum was replaced by serum-free cultured medium before transfection. After successful transfection, Puro was used to screen positive cells.

### Wound-healing assay and transwell assay

Wound-healing assay and transwell assay were performed to detect the change of migration and invasion capabilities of HepG2 and Huh7 cells, respectively. In wound-healing assay, HepG2 and Huh7 cells in a logarithmic growth phase were collected to seeded in a 6-well plate. Subsequently, a wound was created mechanically using a 1 ml pipette tip. In transwell assay, the basement membrane of upper chamber was covered using ABW® Matrigengel (082703, ABW, China).

### Western blotting

Western blotting was carried out according to the manufacturer’s instructions. The antibodies used for western blotting included iNOS (20,609 S, CST, Boston, MA, USA), Arg1 (93,668 S, CST), TREM1 (11791-1-AP, proteintech, Chicago, IL, USA), CCL7 (81,559 S, CST), E-cadherin (3195 S, CST), N-cadherin (13,116 S, CST), Vimentin (5741 S, CST), β-actin (4970 S, CST).

### Human cytokine array

Human Cytokine Array was implemented to detect the main responsive cytokine following with TREM1 knockdown in THP1-derived TAMs. Human Cytokine Array C5 kit (AAH-CYT-5-2, RayBiotech, Inc., Guangzhou) was carried out according to the manufacturer’s instructions.

### ELISA assay

MCP-3/CCL7 Human ELISA Kit (BMS2125, Invitrogen, USA) was implemented to detect the concentration of CCL7 in cells conditional medium. The detailed experimental procedure are as follows. First, 100ul each gradient standard, sample to be measured and sample diluent were correspondingly added to the standard hole, sample measurement hole and blank hole. Measuring plate was coated and subsequently incubated at 37 °C for 90 min. Then each reaction hole was treated with biotin-antibody working solution, HRP-Streptavidin, TMB chromogenic substrate, and reaction stop solution in turn. Cleaning each reaction hole using washing buffer was required after each step completed. Finally, the OD450 value were read for calculating the CCL7 concentration of the sample.

### Statistical analyses

Statistical analyses in this study were carried out using the SPSS 24.0 software (Abbott Laboratories, Chicago, USA). Data were shown as mean ± SD. Grade data were tested for differences between groups using Kruskal-Wallis method. Log-rank test is used for the Kaplan-Meier survival analysis. *P*-values less than 0.05 was identified to be statistically significant.

## Results

### TREM1 highly expresses in metastatic hepatocellular carcinoma and is strongly associated with poor prognosis of patients with hepatocellular carcinoma

**Fig. 2:**
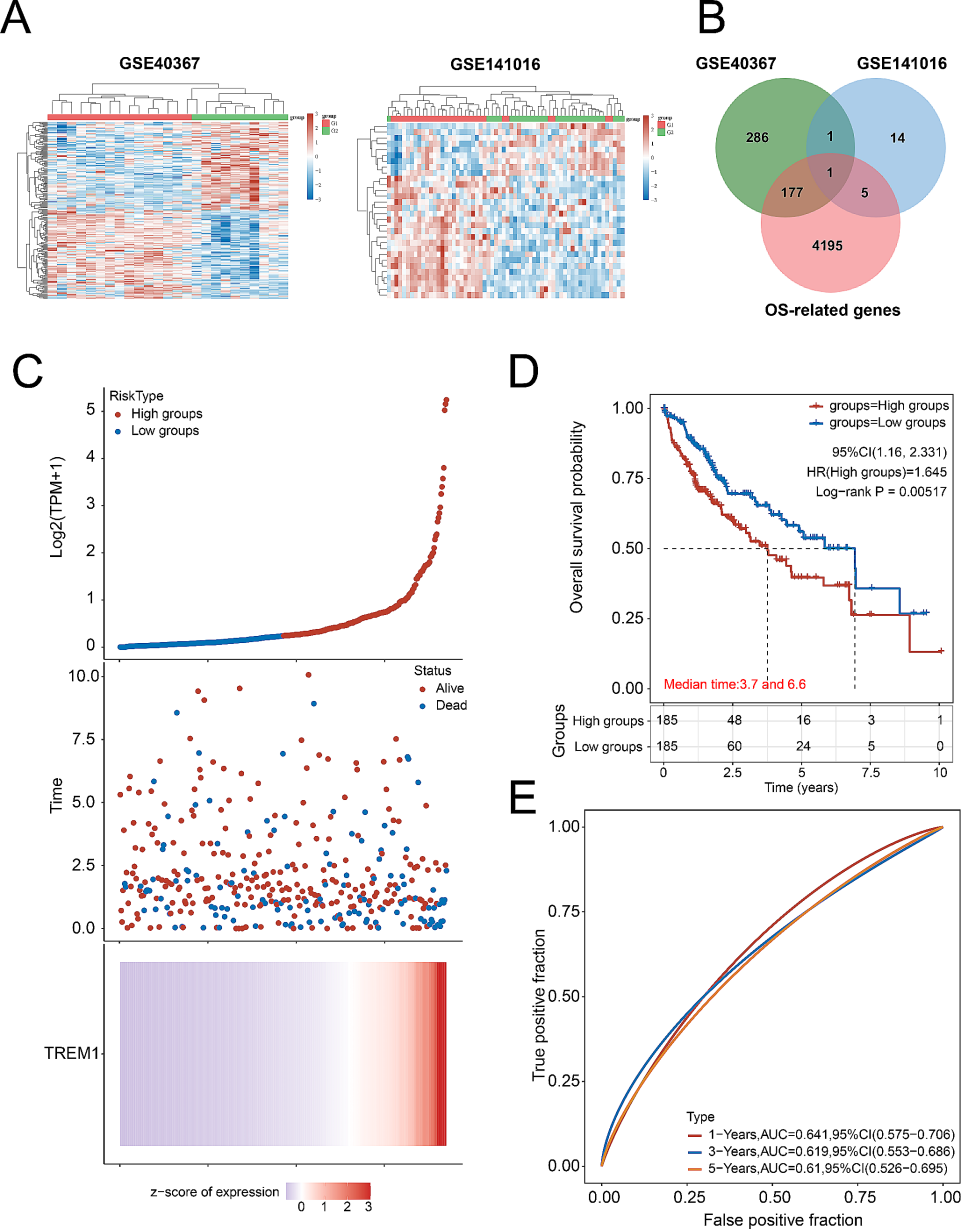
TREM1 was positively associated with poor prognosis of metastatic HCC. **A**. Heat map, the differential gene expression in GSE40367 and GSE141016, G1: primary hepatocellular carcinoma; G2: metastatic hepatocellular carcinoma. **B**. Venn diagram, the intersection gene set of differential gene expression in GSE40367 and GSE141016, as well as positively related to Overall Survival (OS) of HCC. **C**. The relationship between TREM1 gene expression and survival time, survival status of patients with HCC; the top diagram represents the scatter plot from low to high expression level of TREM1; red colors represent high expression groups, and blue colors represent low expression groups; the middle scatter diagram represents the distribution of survival time and survival state corresponding to gene expression in different samples; the bottom diagram represents the heat map of TREM1 expression; **D**. Overall survival analysis of high TREM1 expression group and low TREM1 expression group. **E**. AUC-ROC curves analysis of the accuracy of TREM1 expression predicting 1-, 3-, and 5-year survival rate

To probe the key responsive gene associated with the metastasis and poor prognosis of patients with HCC, we analyzed the intersection of high-expression genes in metastatic HCC and OS-related genes obtained from GEO database. Heat map showed the differentially expressed genes (DEGs) of the patients with metastatic HCC (Fig. < link rid="fig2”>2</link>A and S1-2 Table). The main candidate gene is TREM1 which highly expressed in metastatic HCC and strongly associated with poor prognosis of patients with HCC (Fig. 2B and S1-4 Table). More importantly, we found that TREM1 expression strongly affect the prognosis in patients with HCC using the data obtained from TCGA database. Highly expression of TREM1 was positively associated with poor prognosis in patients with HCC (Fig. 2C-D). Moreover, constructing a model based on the expression of TREM1 can effectively predict the prognosis of patients with HCC (AUC: 0.61–0.641, Fig. 2E). Taken together, TREM1 was highly expressed in metastatic HCC and strongly associated with poor prognosis in patients with HCC.

### TREM1 expression is positively correlation with the infiltration of macrophages in hepatocellular carcinoma microenvironment

**Fig. 3:**
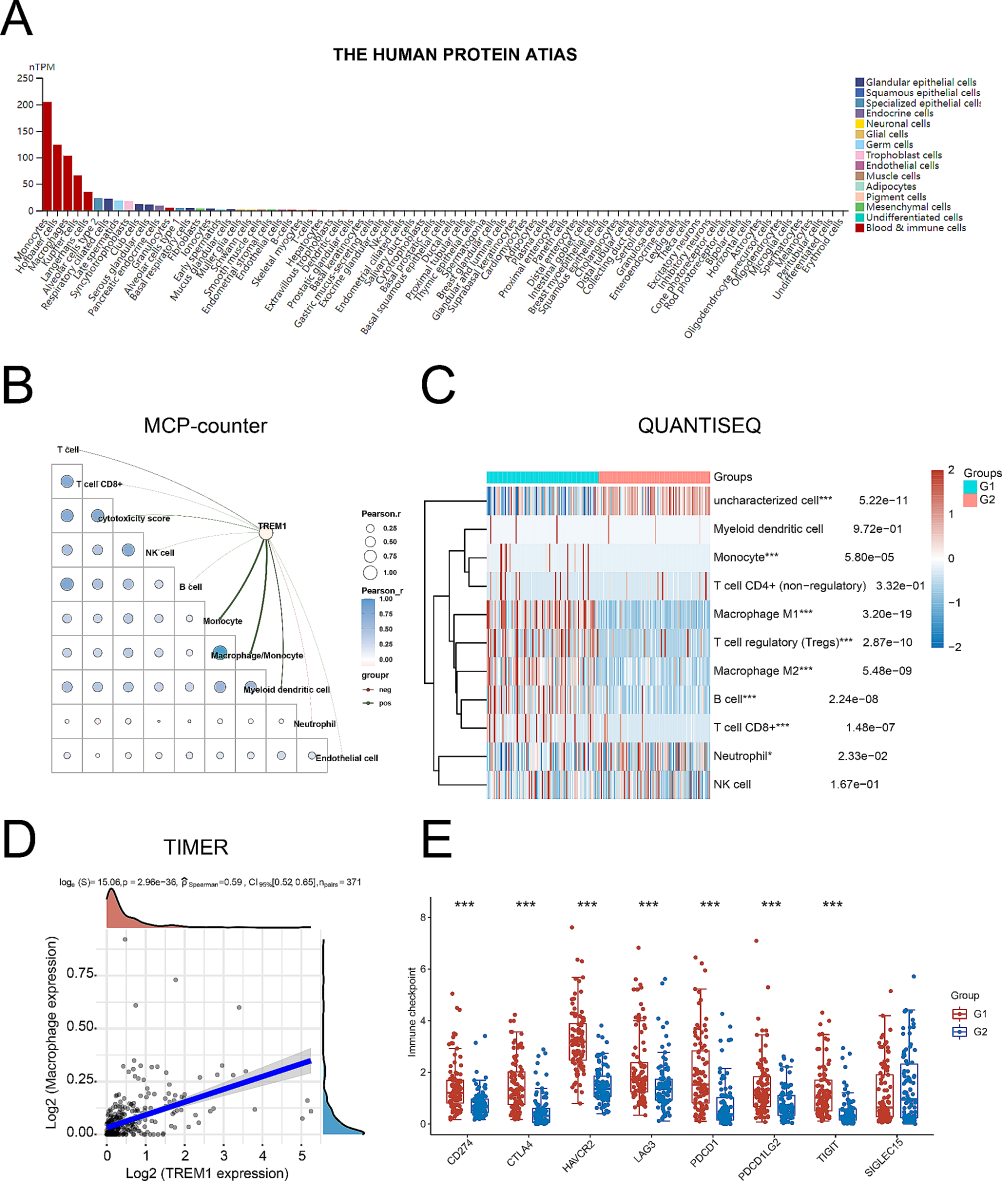
The correlation between TREM1 expression with the infiltration of macrophages in HCC microenvironment. **A**. TREM1 expression in several cell type. **B**. The diagram represents the correlation between TREM1 expression and immune score. Line thickness represents the strength of the correlation, with red representing positive correlation and blue representing negative correlation. Likewise, the circle also represents the strength of the correlation. **C**. Distribution of immunoinfiltration scores with high TREM1 expression and low TREM1 expression; G1 groups as high TREM1 expression, and G2 groups as low TREM1 expression. **D**. The correlation between TREM1 expression and macrophages immunoinfiltration scores. **E**. The correlation between TREM1 expression and several immune checkpoint expressions; G1 groups as high TREM1 expression, and G2 groups as low TREM1 expression

To understand the tumor-promoting effect of TREM1 in HCC, we explored the relationship between TREM1 expression and immune infiltration in HCC. We found that TREM1 was mainly expressed by monocytes in humans based on the data obtained from *THE HUMAN PROTEIN ATIAS* (Fig. 3A). Subsequently, we analyzed the correlation between TREM1 expression and common immune cells in the microenvironment using an MCP-counter method. Interestingly, TREM1 expression was positively associated with the infiltration of macrophages in HCC (Fig. 3B). Moreover, other analysis methods also showed that TREM1 expression mainly caused high infiltration of macrophages in HCC microenvironment (Fig. 3C-D). It is obvious that macrophages as the main cell population response to immunosuppressive microenvironment. Hence, we also demonstrated that TREM1 expression was positively correlation with the expression of several immune checkpoint including CD274, CTLA4, HAVCR2, LAG3, PDCD1, PDCD1LG2, and TIGIT (Fig. 3E). In conclusion, TREM1 is mainly expressed by macrophages and is positively correlated with tumor immune escape.

### Sing-cell RNA sequencing analysis reveals that TREM1 mainly expressed by monocytes

**Fig. 4:**
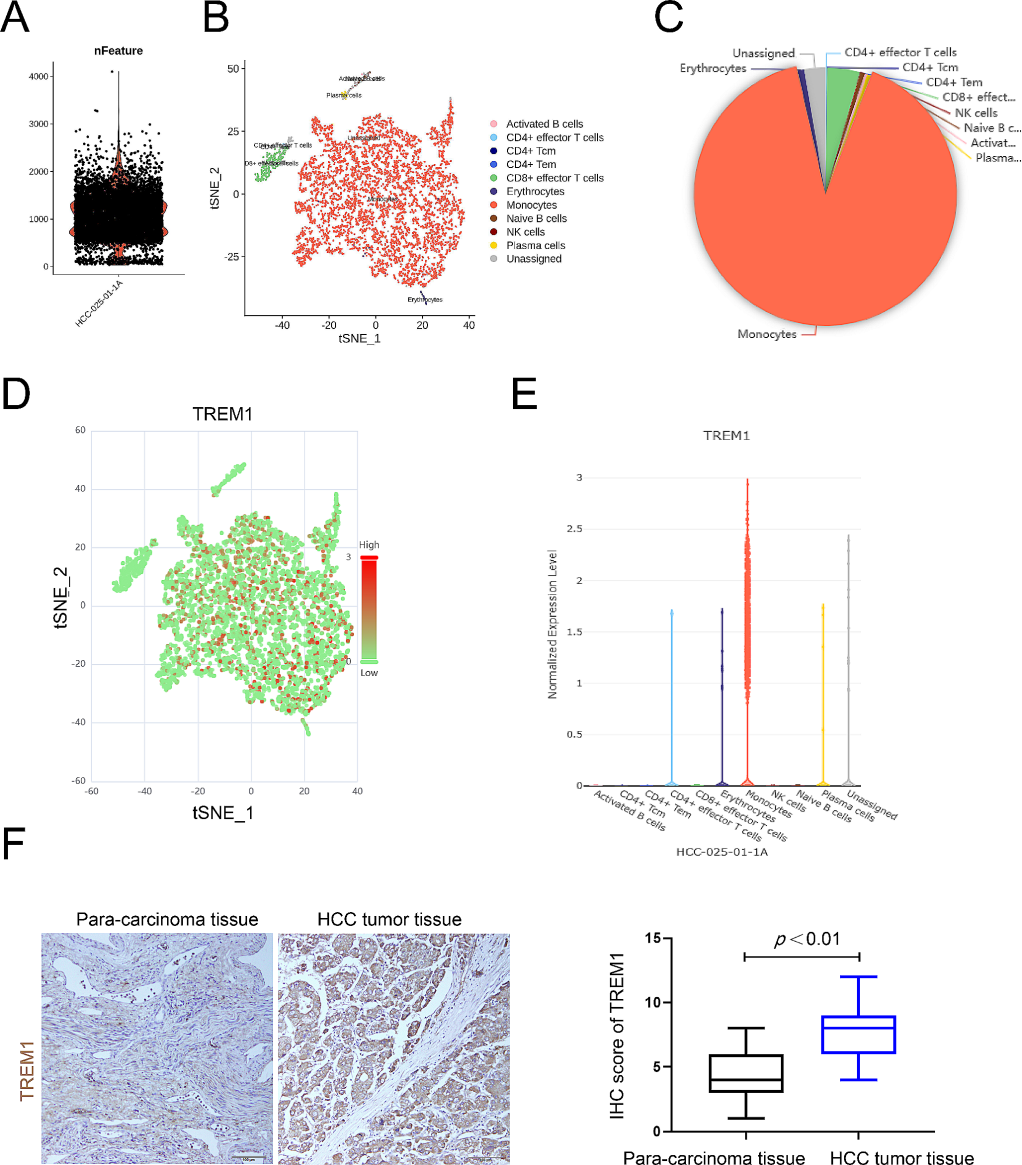
Sing-cell RNA sequencing analysis of hepatocellular carcinoma tissue. **A**. The genetic data detected in each cell; The Y axis represents the number of genes. **B**. The results of clustering using tSNE method, and different colors represent different cell groups. **C**. Statistical map of the difference in the content of different cell types. **D**, **E**. the TREM1 expression in specific cell type. **F**. tissue microarray analysis of TREM1 expression in HCC tissue, *p < 0.05, **p < 0.01

Single-cell RNA sequencing was used to identify the main cells expressing TREM1 in HCC microenvironment. Required data was download from GEO database and analyzed using *CancerSCEM* web-based tools. After performing Quality Control and Cluster Identification of these single-cell sequencing data, it was found that monocytes accounted for the largest proportion in HCC microenvironment (Fig. 4A-C). On further analysis, it was found that TREM1 mainly expressed by monocytes in HCC microenvironment (Fig. 4D-E). Moreover, the previous results showed that TREM1 expression was positively related to the high infiltration of macrophages in HCC microenvironment (Fig. 3B-D). In order to evaluate the expression of TREM1 in liver cancer tissues, a tissue microarray was used to detect TREM1 expression. The results revealed a significant upregulation of TREM1 in liver cancer tissue (Fig. 4F). Taken together, TREM1 mainly expressed by immune cells especially by macrophages in HCC microenvironment.

### TREM1 knockdown abrogates the effect of TAMs in promoting the metastasis and EMT of hepatocellular carcinoma cells

**Fig. 5:**
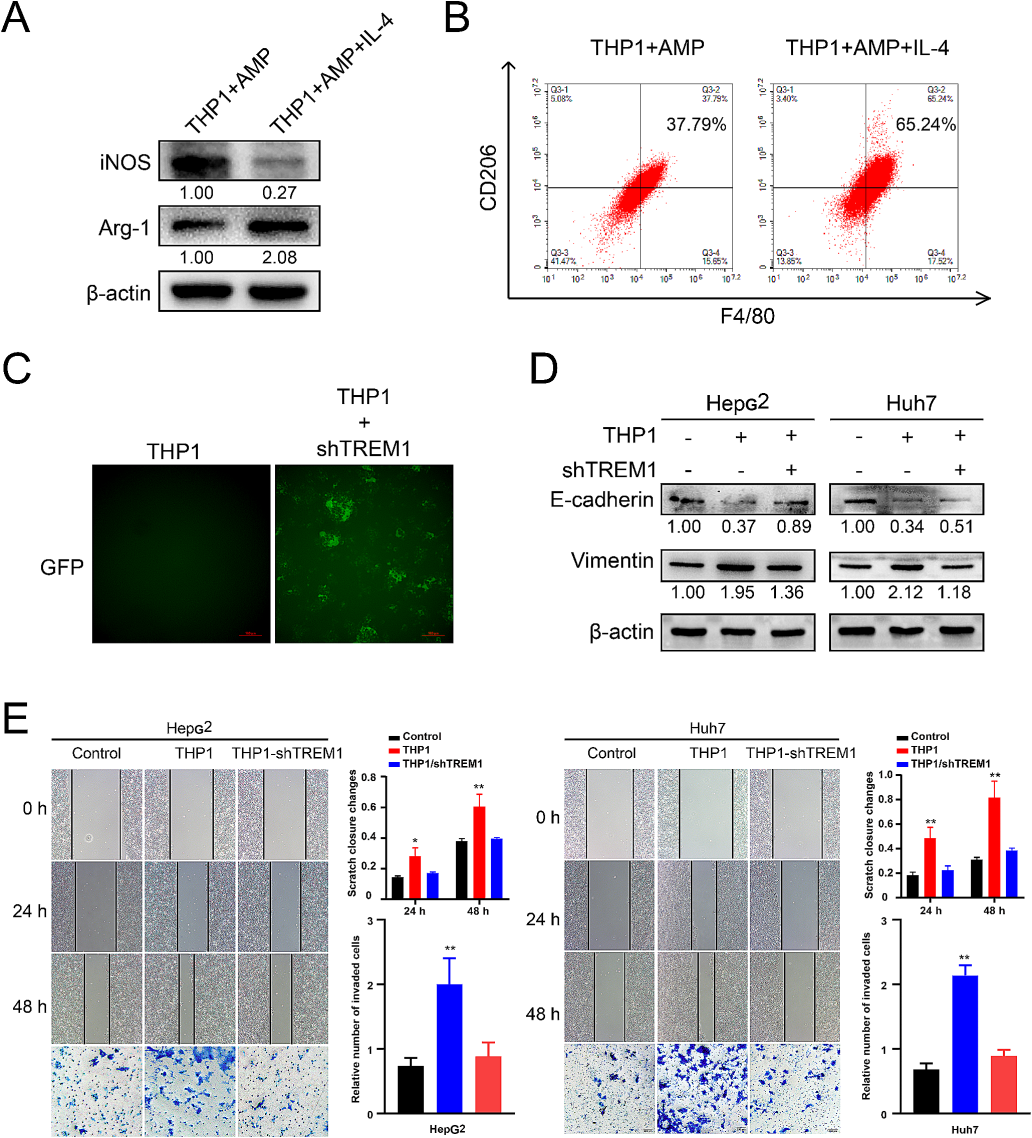
The effect of TREM1 knockdown in THP1-derived TAMs. **A**, **B**. The verification of THP1-derived TAMs was analyzed via the macrophage surface markers. THP1 monocytes were induced differentiation into macrophages (M0) by 100 ng/ml PMA treatment for 24 h, subsequently, 10 ng/ml IL-4 was used for the differentiation of M2 subtype TAMs. **C**. The reliability of TREM1 knockdown in THP1 monocytes was validated; the plasmid of shTREM1 is pLent-U6-shRNA-CMV-copGFP-P2A-puro. **D**, **E**. the conditioned medium of THP1-derived TAMs significantly promoted the EMT, migration and invasion of Hepatocellular carcinoma cells, while TREM1 knockdown abrogated that. All values are presented as the mean ± SD. *n* = 3, *p < 0.05, **p < 0.01

Uncovering the pro-oncogenic effects of TREM1^+^ TAMs might provide potential therapeutic target for metastatic HCC. Therefore, we further investigated whether manipulating the TREM1 expression of TAMs can affect the metastasis of HCC cells. In the present study, tumor-associated macrophages induced by THP1 monocytes in vitro have been verified as a viable method(Huang et al. 2020). THP1 monocytes can differentiate into macrophages (M0) using PMA stimulation. Subsequently, IL-4 stimulation induced M0 macrophages differentiate into M2 macrophages. Specifically, THP1-derived macrophages (TAMs) embodied the high expression of CD206 marker and Arg-1 marker (Fig. 5A-B). Plasmid transfection was used to knock down the TREM1 expression of THP1 monocytes (Fig. 5C). The conditioned medium obtained from THP1-derived TAMs can promote the migration and invasion of HCC cells, while TREM1 knockdown partly abrogated that (Fig. 5E). Interestingly, we found that the conditioned medium obtained from THP1-derived TAMs significantly promoted the EMT of HCC cells which manifest as the decreased expression of E-cadherin and the increased expression of Vimentin. Similarly, TREM1 knockdown partly abrogated that (Fig. 5D). The results suggested that TREM1 knockdown abrogated the pro-oncogenic effect of TAMs. Taken together, TREM1^+^ TAMs can promote the metastasis and EMT of HCC cells.

### TREM1^+^ TAMs secrete CCL7 to promote the metastasis and EMT of hepatocellular carcinoma

**Fig. 6:**
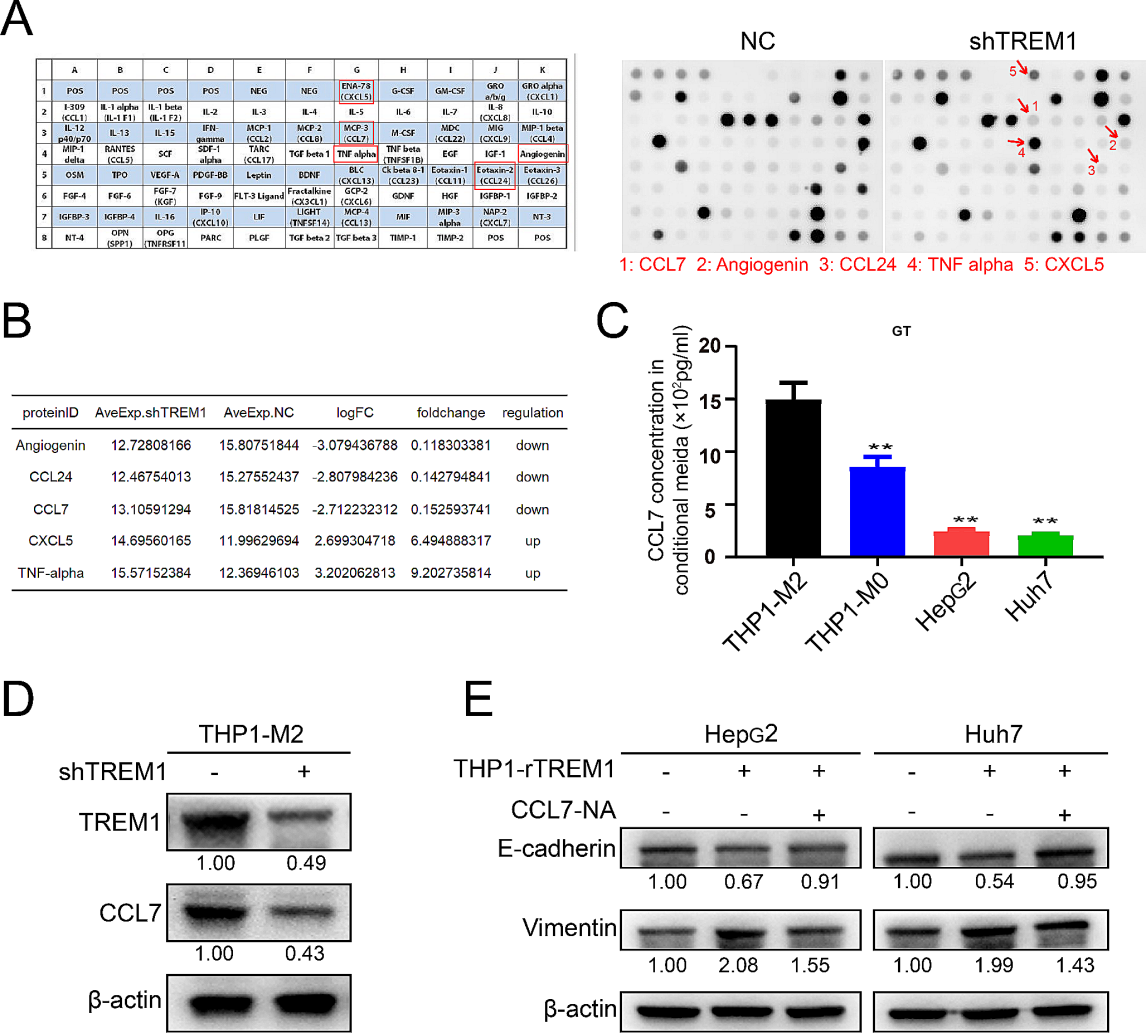
CCL7 derived from TREM1^+^ tumor-associated macrophages promote hepatocellular carcinoma metastasis. **A**, **B**. The differential expression cytokine of TREM1 manipulation in THP1-derived TAMs was detected using Human Cytokine Array. **C**. The concentration of CCL7 in condition medium of M2 subtype TAMs, M0 subtype TAMs, and hepatocellular carcinoma cells was detected. **D**. TREM1 knockdown inhibited the CCL7 expression in THP1-derived TAMs. **E**. The condition medium of THP1/rTREM1-derived TAMs can promote the EMT of hepatocellular carcinoma cells, while CCL7-NA abrogated that. All values are presented as the mean ± SD. *n* = 3, *p < 0.05, **p < 0.01

To investigate the underlying mechanism of TAMs on the metastasis of HCC, a cytokine array provided by *Raybiotech* company was used to probe the main cytokine in condition medium of TREM1-knockdown TAMs. The result shows that CCL7 was the key cytokine response to TREM1 manipulation, which suggested TREM1^+^ TAMs may secrete CCL7 to promote metastasis of HCC (Fig. 6A-B). Obviously, CCL7 was most high expression in condition medium of M2-TAMs (Fig. 6C). Moreover, TREM1 knockdown in THP1-derived TAMs can decreased the expression of CCL7 (Fig. 6D). The commercialized recombinant human CCL7 neutralizing antibody (NA) (MAB282, R&D Systems) was used to reduce the concentration of CCL7 in condition medium. The rescue experiment results suggested that the overexpression of TREM1 in THP1-derived TAMs can promote the EMT of HCC cells, accompanied by increased expression of Vimentin and the decreased expression of E-cadherin; and CCL7-NA can partly abrogate that (Fig. 6E). Taken together, TREM1^+^ TAMs can secrete CCL7 to promote the metastasis and EMT of HCC.

## Discussion

Hepatocellular carcinoma is the sixth most common cancer and the third leading cause of cancer-related death in 185 Countries(Sung et al. 2021). The prognosis of early hepatocellular carcinoma is good, and radiofrequency ablation and surgery are the common treatment strategies(Kulik and El-Serag 2019; Brown et al. 2023). When the disease develops to the advanced stage, there are very few treatment strategies can be chosen. The complicated etiological mechanism of hepatocellular carcinoma leads to the specificity and complexity of its tumor microenvironment. Hepatocellular carcinoma lacks driver genes that can be used to develop molecularly targeted therapies, so immune-based therapies is an effective therapeutic strategy for hepatocellular carcinoma(Dong et al. 2020; Hao et al. 2021; Greten et al. 2019). Accumulating evidence suggest that high infiltration of tumor-associated macrophages can significantly promote the metastasis of hepatocellular carcinoma (Zheng et al. 2023; Lu et al. 2023; Xu et al. 2022a, b; Li et al. 2019; Huang et al. 2023). Therefore, it is very important to carry out in-depth analysis of the pro-oncogenic mechanism of tumor-associated macrophages for improving the clinical therapeutic effect of hepatocellular carcinoma. Although there are many studies have examined the mechanisms and identified the pro-oncogenic effects of tumor-associated macrophages, the therapeutic efficacy of direct elimination strategies against tumor-associated macrophages in vivo has been disappointing. This may be due to the heterogeneous tumor microenvironment networks and multimolecular typing of tumor-associated macrophages.

As the key components of tumor microenvironment, TAMs attract more and more researchers’ attention. The traditional classification methods simply divide TAMs into two subtypes: M1 subtype and M2 subtype. With the deepening of research, it is found that the simple dichotomy is difficult to accurately characterize the function of TAMs. For example, after operating TACE of hepatocellular carcinoma, TREM2^+^ macrophages inhibit CD8^+^ T cells infiltration which causes the recurrence and progression of HCC(Tan et al. 2023). PPT1^+^ macrophage infiltration is associated with poor prognosis of patients with HCC, and targeting PPT1 can enhance the immunotherapy efficacy of HCC(Weng et al. 2023). Identifying these specific pro-oncogenic molecules expressed by TAMs will help us develop strategies of targeted clearance of TAMs and improve the clinical therapeutic outcomes of patients with HCC.

The myeloid trigger receptor (TREM) family is an important family of receptors specifically expressed in myeloid cells and involved in many functions of myeloid cells. At the same time, they are also widely expressed on the surface of natural killer cells, B cells, T cells, epithelial cells and endothelial cells, indicating the importance of regulating innate immunity(Juric et al. 2023). A study has shown that TREM1 is highly expressed in hepatocellular carcinoma tissue and significantly promotes the proliferation and invasion of HCC cells(Duan et al. 2015). However, the specific immune cell localization of TREM1 in hepatocellular carcinoma tissue needs further study. Hence, to solve this problem, we performed a single-cell RNA sequencing analysis and found that TREM1 was mainly expressed by monocytes. In addition, many studies have demonstrated that TAMs can secrete multiple cytokines to promote tumor metastasis(Wang et al. 2021). Chemokines are a large family of small cytokines with molecular weights ranging from 7 to 15 kDa(Huang et al. 2021). TAM-derived chemokines have been reported to be associated with cancer progression and metastasis. For example, a study reported that tumor-associated macrophages secrete TGF-β1 to promote cancer stem cells and EMT in hepatocellular carcinoma(Fan et al. 2014). Multiple studies have shown that CCL7 plays an important role in cancer metastasis, such as promoting the metastasis of breast cancer(Kanyomse et al. 2022), colorectal cancer(Xu et al. 2022a, b), gastric cancer(Chen et al. 2020) and other tumors. In particular, cancer-associated fibroblasts in HCC tumor tissue can secrete CCL7 to promote HCC metastasis(Liu et al. 2016). Accumulating evidence identify CCL7 as a key pro-metastasis cytokine, so this study focuses on CCL7 function. Herein, we found that CCL7 was highly expressed in M2-TAMs condition medium, and lowly expressed in the culture medium of TAMs with TREM1 knockdown. Further rescue experiment results suggested that TREM1 overexpression in THP1-derived TAMs can promote the EMT of HCC cells. Taken together, TREM1 and CCL7 are the proven cancer-promoting factors of hepatocellular carcinoma.

## Conclusions

In summary, the present study demonstrates that TREM1^+^ TAMs are strongly correlation with poor prognosis in patients with HCC via promoting EMT. This study not only uncovers the mechanism of TREM1^+^ TAMs in promoting the metastasis of HCC but also provides a novel rationale for developing TREM1/CCL7 as a potential molecular target for the treatment of metastatic HCC.

### Electronic supplementary material

Below is the link to the electronic supplementary material.

Supplementary Material 1

Supplementary Material 2

Supplementary Material 3

Supplementary Material 4

## Data Availability

No datasets were generated or analysed during the current study.
